# Linking physiological state to movement dynamics in an open ocean predator, the blue shark (*Prionace glauca)*

**DOI:** 10.1371/journal.pone.0337589

**Published:** 2026-01-07

**Authors:** Austin J. Gallagher, Evan B. Byrnes, Rachel A. Skubel, Brian Raymond, Joe Romeiro, Steven J. Cooke, Garrett M. Street, Neil Hammerschlag

**Affiliations:** 1 Beneath The Waves, Boston, Massachusetts, United States of America; 2 Centre for Sustainable Aquatic Ecosystems, Harry Butler Institute, School of Science, Health, Engineering and Education, Murdoch University, Murdoch, Washington, Australia; 3 Abess Center for Ecosystem Science and Policy, University of Miami, Miami, Florida, United States of America; 4 Pelagic Expeditions, Point Judith, Rhode Island, United States of America; 5 333 Productions, Coventry, Rhode Island, United States of America; 6 Fish Ecology and Conservation Physiology Laboratory, Department of Biology, Carleton University, Ottawa, Canada; 7 Quantitative Ecology & Spatial Technologies Laboratory, Mississippi State University, Starkville, Mississippi, United States of America; 8 Rosenstiel School of Marine and Atmospheric Science, University of Miami, Miami, Florida, United States of America; James Cook University, AUSTRALIA

## Abstract

The movement behavior of open ocean fishes is challenged by metabolic demands resulting from sustained swimming and the availability of resources in a dynamic, ephemeral environment. Advances in electronic tagging and tracking technologies have permitted unprecedented opportunities to describe the movements of open ocean fishes in these environments, however, our understanding of the mechanistic drivers of individual variation in movement performance is limited. In this study, we tested the hypothesis that movement capacities of open ocean sharks would be related to physiology and body condition. We measured the physiological status (e.g., energy stores and body condition) of blue sharks (*Prionace glauca*) captured in the open ocean, and then tracked their movements over 45 days. We then explored for relationships between physiological metrics and individual differences in metrics of movement behavior (distance traveled, activity space, behavioral state, and tortuosity) of the tracked blue sharks. Analyses detected consistent positive relationships between individual plasma triglyceride concentrations and body condition (sampled at time of capture) on distance traveled, activity space, behavioral state, and tortuosity - up to 45 days post tagging, with models explaining up to 79% of individual variation in movement. These findings highlight the potential role of metabolic lipid reserves in shaping movement behavior of open ocean, predatory sharks in patchy, ephemeral environments. More broadly, they offer new insight into the factors which may influence individual variation in the timing and scale of movement in oceanic fishes.

## Introduction

Electronic tagging has transformed the research community’s ability to track and describe the movements of highly mobile oceanic predators [1,2,3], capturing large-scale displacements, fine-scale movement patterns, and variation among individuals in space use and performance [e.g., 4,5,6]. These datasets have also been collated, synthesized, and integrated with big data approaches to inform conservation and management at ocean-basin scales [7,8]. While these advances have revealed remarkable complexity in movement behavior, the mechanisms underlying such individual differences remain poorly understood [9].

A comparative study of movements across different species of marine megafauna found that displacement, a daily measure of performance, was generally not affected by body length or mass [10], but instead, that complex movements were more related to microhabitat complexities which were hypothesized to be linked to nutritional or foraging-related benefits. Interestingly, work utilizing high-resolution tracking demonstrated that large predatory shark species such as the white shark (*Carcharodon carcharias*) and blue shark (*Prionace glauca*) seek out mesoscale oceanographic features known as eddies, where temperature anomalies create slightly warmed conditions and presumed access to energetically profitable prey [11–13]. These studies provided new insights into the importance of biological and physical features, which may influence dynamic animal movement in the open ocean.

Energy acquisition plays a major role in the movement ecology of fishes (reviewed in [14], and a horizon scan of the top 100 most-pressing questions for understanding the future of fish migration/movement, conservation, and policy identified the concept of internal state as the very first item on the list: “What are the internal physiological drivers of, and triggers for, migration?” [15]. While the theoretical link between animal movement and energy acquisition is well understood, studies integrating measures of internal physiology with movement are surprisingly lacking. However, there are a suite of non-invasive tools available to researchers to obtain small tissue biopsies (e.g., blood or muscle sample) from tagged animals, providing an opportunity to explore relationships between aspects of organismal condition (e.g., physiological status, health, gene expression) and their subsequent behavior and fate [1]. Furthermore, certain physiological endpoints assayed from blood plasma can be used as biomarkers for relevant questions related to energy use, metabolism, stress, and even prey quality [16–19]. One of the first studies to adopt this approach was on adult Pacific salmon (*Oncorhynchus* spp.), demonstrating physiological correlates of extensive movement behavior and premature mortality, thus revealing a mechanistic basis between movement behavior and fate [20].

Sharks generally store large quantities of fats in their liver, which can constitute a significant proportion of their body mass [21]. These reserves play a central role in lipid metabolism during extended movements and throughout parturition [19,22,23]. Triglycerides (TAG, as abbreviated in the present study) from the diet are conservatively transferred to heptic storage [24,25], and the low density of the liver relative to seawater also provides lift, thereby reducing locomotory costs [26]. Ketone bodies are recognized as a primary fuel during activity, but plasma triglycerides (TAG) remain a widely used and accessible proxy for long-term energy stores because they can be readily quantified from small blood samples in sharks [17] and have been linked to overall energetic condition in several species [16,18,27,28]. While the lipid metabolism of sharks is a complex and a somewhat limited area of study [16,17,29], these dynamics highlight the importance of the liver in both energy storage and movement ecology, and thus the value of examining lipid metabolites such as TAG in migratory sharks.

In the present study, we investigated the potential physiological and behavioral correlates of intra-specific differences in movement of an oceanic predator, the blue shark, over short time sales (up to 45 days). Operating under the hypothesis that variability in movement may be related to differences in physiological state [30], we explored whether individual differences in distance traveled, activity space, behavioral state, and tortuosity of tagged blue sharks were correlated with plasma triglyceride levels (a measure of endogenous energy reserves), body condition (a proxy of overall health), and shark body length. Blue sharks are a useful oceanic species to examine these patterns as they are known to target open-ocean ephemeral features, like mesoscale eddies, along ocean frontal zones, where they are believed to feed [12,13].

## Materials and methods

### Capture, body measurements and physiological sampling

**Fig 1:**
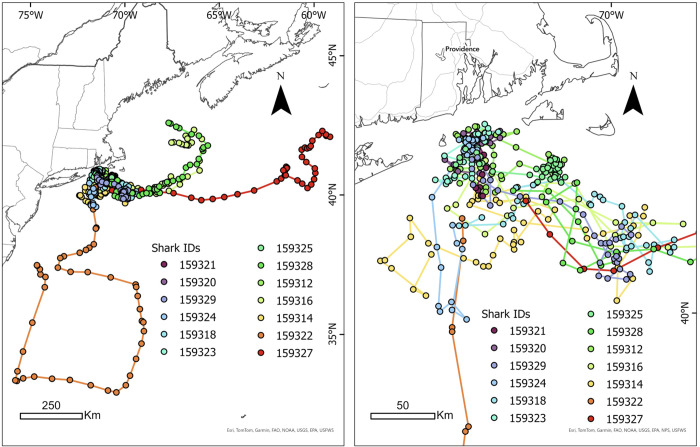
Map of the study area off the United States Eastern seaboard, with interpolated tracks and total distance traveled by the 13 male blue sharks tagged in the present study. The IDs of tracks are ordered and color coded by track length as follows: track length increases from IDs ordered along the blue to red color range as identified in the figure legend. Similarly, IDs listed earlier in the legend have relatively shorter tracking durations (colder color palate) and those ordered towards the end of the list have longer tracking durations (warmer color palate). Left display shows broader latitudinal range, whereas the right panel is zoomed in on those tracks from the core area off New England. Map developed in ESRI ArcGIS 10.3.3. Reprinted from Esri under a CC BY license with permission from Esri, original copyright 2016.

Blue sharks were captured using baited circle-hook handlines in federal waters off Point Judith, Rhode Island, USA (41.3615 N, −71.48139 W) from June 1 – September 1, 2016 (Table 1, Fig 1], overlapping with their seasonal offshore aggregation in the region [31]. All animal tagging and capture was ethically approved by the Carleton University Animal Care Committee. Sharks were immediately secured on a platform inside the boat and a saltwater hose and pump was inserted in the shark’s mouth. Each shark was measured for total length (TL in cm) and an additional five measurements were taken to estimate body condition based on a Span Condition Analysis (SCA) following Irschick and Hammerschlag [32]. These measures included: (1) pre-caudal length [PCL], the distance from the tip of the shark’s snout to the caudal peduncle; (2) lateral span [LS], the distance spanning across the surface of the shark between the anterior insertion points of the pectoral fins; (3) frontal span [FS], measured at the anterior insertion point of the first dorsal fin, the distance spanning the surface of the shark between lines parallel to the frontal plane that extend from the pectoral fins to the caudal fin; (4) proximal span [PS], measured at the posterior insertion point of the first dorsal fin, the distance spanning the surface of the shark between lines parallel to the frontal plane that extend from the pectoral fins to the caudal fin; and (5) caudal keel circumference [CKC], measured at the caudal peduncle, the circumference at the base of the caudal fin. From these measurements, the body condition of each shark was calculated, using the SCA equation: SCA = Σ (LS + FS + PS + CKC)/(PCL). Larger SCA values are suggestive of higher body condition, whereas lower SCA values confer a poorer condition [32].

**Table 1:** Summary of animals and tagging info for the individual blue sharks tracked and analyzed in the present study.

SATID	Date Tagged YYYY-MM-DD	Sex	Total Length (cm)	Release Latitude	Release Longitude	Days At Liberty*	Distance Traveled (km)**	TAG (mmol l-1)	Condition
159314	2016-06-15	M	240	40.79	71.38	63	785.35	0.35	0.99
159322	2016-06-15	M	254	40.79	71.38	67	2149.84	0.95	0.94
159328	2016-06-30	M	281	40.79	70.8	46	1123.15	0.91	0.99
159329	2016-07-06	M	288	40.9	71.27	29	312.66	0.12	0.89
159316	2016-07-07	M	262	40.88	71.2	51	1438.95	0.15	1.09
159327	2016-07-07	M	254	40.88	71.2	84	1368.16	0.08	1.07
159318	2016-07-12	M	313	40.89	72.19	33	387.04	0.03	0.93
159321	2016-07-12	M	269	40.89	72.19	11	75.35	0.05	0.97
159320	2016-07-27	M	269	40.13	71.01	22	191.24	0.02	0.96
159323	2016-07-27	M	265	41.13	71.09	66	315.37	0.18	0.96
159325	2016-07-27	M	239	41.13	71.09	38	319.13	0.19	1.02
159312	2016-08-07	M	266	41.01	71.38	47	692.53	0.21	0.96
159324	2016-08-15	M	240	41.12	71.04	32	352.38	0.46	0.94

*Total days tracked from first to last transmission.

**Distance clipped to maximum fitted 5-day time bin (0–45 days).

A 7mL blood sample was taken via caudal venipuncture on un-anesthetized sharks [33]. Whole blood was immediately spun down on the boat (5000 rpm for 4 minutes) and the plasma was then separated into three 2 mL vials and kept in a water-ice slurry for up to 8 hours before being transferred to a −20°C freezer back on land [34]. We ran metabolic assays on the frozen plasma samples to quantify levels of total plasma triglycerides [free and bound to lipoproteins] (TAG; mmol l^–1^, EnzyChrom Triglyceride Assay Kit, BioAssay Systems, Haywood, CA, USA) on a 96-well microplate absorbance reader at 570 nm (Tecan Sunrise, Tecan, Austria). We chose TAG because fatty acids have been suggested to serve as a useful metric for evaluating shark condition and energy over time [16,18,27,28], and the nature of the metabolite in sharks is such that its presence in the plasma is derivative from stored endogenous reserves [29]. While the exact turnover rate from feeding to TAG storage is unknown in sharks, the short-term time period of our study (i.e., + /- 1 month) was chosen such that the internal TAG values were likely reflective of recent feeding events along the same temporal scale (e.g., the time frame from when exogenous energy will become metabolically visible in blood chemistry; [25]). Concentrations of TAG values were determined using the appropriate standard curves. Inter-assay variation was 2.0%.

### Satellite tagging and spatial data treatment

Prior to release, each shark was tagged with a Smart Position and Temperature Transmitting tag (SPOT6, Wildlife Computers). Tags were mounted to the shark’s first dorsal fin using titanium bolts, steel washers and high carbon steel nuts following Hammerschlag et al. [35]). Geographic locations of SPOT tagged sharks were determined by Doppler-shift calculations made by the Argos Data Collection and Location Service (Argos CLS; www.argos-system.org). Locations were acquired when the tag broke the water surface and transmitted a signal to passing Argos satellite. To improve location accuracy, all Doppler derived data were processed automatically by Argos CLS using Kalman filtering (KF). Argos provides location accuracy using location classes (LC) 3, 2, 1, 0, A, B, and Z (in decreasing accuracy), corresponding with the following error estimates: LC3 < 250 m, 250 m < LC2 < 500 m, and 500 m < LC1 < 1500 m. The error estimates associated with LC A and B are reported to be > 1 km and >5 km, respectively [36]. LC Z estimates are inaccurate or unreliable and were removed from the dataset prior to any analysis.

Due to irregular surfacing of sharks (and thus irregular transmission rates) and variation in satellite coverage at any given time, raw SPOT-derived data are often subject to data gaps, autocorrelation and spatial biases. Therefore, prior to any spatial analyses, several steps were taken to alleviate these potential issues, including track interpolation and regularization [see 6]. To prevent interpolating between positions with large temporal gaps, individual tracks with data gaps of more than five days between subsequent positions and/or individual tracks with less than 10 positions were split into separate track segments. Next, a hierarchical, first-difference, correlated, random-walk, state-space switching model [SSM; 37,38] was applied to these data following [39]. Specifically, position data were interpolated and regularized to daily estimates in the R statistical software package ‘bsam.’ This package also applied a speed filter of 5 m/s. All subsequent movement analyses were conducted on the interpolated, regularized positions estimates from the SSM, hereafter referred as position relocations.

### Movement performance metrics

We calculated four metrics of movement behavior for each shark: distance travelled (km), behavioral state, tortuosity, and activity space (km^2^). Behavioral state and tortuosity were chosen because they have previously been linked to foraging activities. More spatially restricted, convoluted movements are associated with exploiting resources, whereas more directed, transient movements are associated with moving between resource patches. Moreover, switching behavior states may be part of an overall oceanic foraging strategy for species like blue sharks. To allow comparisons among individuals in movement performance over standardized tracking periods and to also permit evaluating potential relationships between biological variables and movement metrics for different tracking durations, all movement performance metrics (including tortuosity and behavioral state) were calculated for cumulative five-day tracking intervals (i.e., 0–5 days, 0–10 days, 0–15 days etc., inclusive). Analysis of movement data over cumulative five-day intervals for the 45-day period was chosen as it aligns with the relative window in which some lipid metabolites are known to continue to increase in sharks following feeding [25]. Movement metrics were not generated beyond 45 days because the satellite tag batteries of most tracked sharks were depleted due to unexpected high rate of transmissions (average 1000 transmissions per day) from blue shark surfacing [40,41]. Despite this technological limitation, this surface behavior likely benefitted the spatial accuracy of onboard tags as they had more time to uplink to Earth-orbiting satellites [42].

Distance traveled was calculated as the total Euclidian distance (km) moved for each 24-hour period, summed over each cumulative five-day tracking interval. Estimates of activity space (km^2^) were calculated for each cumulative five-day tracking interval by determining the area of the smallest polygon that encompassed all position relocations. Metrics of activity space were computed using the Minimum Bounding Geometry tool in ArcGIS 10.3.3. [ESRI 2016]. This metric of activity space provides a simple metric of evaluating space use, without any assumption about habitat preferences. While other approaches for evaluating space use are available, such as kernel density estimates [43], such metrics are used to investigate home range and spatial preferences, which was not the goal of this work. Tortuosity, a measure of complexity in an animal’s track, was calculated here using VFractal [44] to give an estimate of the interpolated track’s turning angle across each discrete tracking interval. Measured between 1 and 2, lower values near 1 suggest more direct path movement, whereas values approaching 2 signify tortuous and complex path movements. For each independent tracking interval, behavioral state – the classification of movement patterns into categories that approximate underlying behaviors – was calculated from data generated from the Bayesian state-spaced model [39], which allows for individuals to switch between two behavioral states: a “resident” state (area-restricted searching, as values approach ‘2’), and a “transient” state (ranging behavior, as values approach ‘1’). The state-spaced model output produced a behavioral state metric for each 24-hour period, with a value ranging on a continuum between 1 (transient) and 2 (resident). Uncertainty in the value of behavioral state at time *T* is quantified with a Markov Chain Monte Carlo (MCMC) algorithm providing values between 1 and 2. For movement analyses, we calculated the mean behavioral state value and tortuosity for each cumulative five-day tracking interval for each shark.

All research activities and were acknowledged and approved under formal Letters of Acknowledgement (LOA) issued by the National Marine Fisheries Service to AJG and NH (SHK-LOA-16). All activities took place in US Federal waters; none of these areas were privately owned nor were they protected under any specific management plans. All research was non-take, and no protected species were sampled. The research and protocols conducted in this study complied with relevant ethical regulations as approved by the Carleton University Animal Care Committee.

### Statistical analyses

We evaluated the relationships between internal state (TAG, body condition, shark size) and movement (distance traveled, rate of movement, activity space, tortuosity, behavioral state) over the entire tracking 45-day tracking duration using a combination of linear (LM) and generalized linear models (GLM). For distance traveled and activity space, we observed that these metrics of animal movement often saturate toward some maximum, typically in a log-linear fashion [e.g., due to home-ranging or otherwise spatially restricted movements; [45,46]. Thus, we structured these LMs such that each log-transformed response variable (distance traveled, activity space) was expected to increase with tracking interval but at diminishing rates following a simple quadratic function (i.e., $ln\hat{Y}=\beta_{\text{0}}+\beta_{\text{1}}t+\beta_{\text{2}}t^{\text{2}}$, where *t* is the time interval since capture). However, our hypotheses posited that the rate of change in the response with time is determined by the physiological and morphological traits of the animals at capture. We thus included first-order interactions between the main linear effect of time and TAG, body condition, and shark size (TL). This parameterization allowed us to specifically ask, after accommodating the known and strong effect of time on our movement metrics: do our physiological and morphological measurements of the animals meaningfully explain the remaining variation in movement metrics? If our hypotheses were correct, we expected to see strong interactions between *t* and TAG, body condition, and TL. We also centered-and-scaled TAG, body condition, and TL such that their respective effects on each response would be directly comparable. This model structure allowed us to evaluate whether TAG, body condition, and TL had any effect on the response variables after controlling for the well-established log-linear relationships between the response variables and time [45]. Additionally, the center-and-scaling allows us to compare the predicted effects on our movement metrics at low (−2 standard deviations from the mean), medium (the mean), and high (+2 standard deviations from the mean) values of each physiological or morphological trait.

Tortuosity values from VFractal and behavioral state estimates from the state-space model are both naturally bounded between 1 and 2. For consistency in modeling, we rescaled both variables to the interval (0,1) by subtracting 1 from all values. We then constructed GLMs for these response variables assuming a beta distribution with a logit link, following the same model structure as for the LMs described above (i.e., logit Ŷ = β₀ + β₁t + β₂t², with first-order interactions between *t* and centered-and-scaled TAG, body condition, and TL).

Diagnostic plots for all LMs and GLMs revealed no violations of model assumptions or overdispersion. VIF scores indicated multicollinearity only for time interval and its square (as expected since one is derived from the other). Models were evaluated with respect to *R*^2^ (LM) or pseudo-*R*^2^ (GLM), with statistical significance declared at p≤0.05. All analyses were performed in R v. 4.0.4 (R Core Team 2021).

## Results

**Fig 2:**
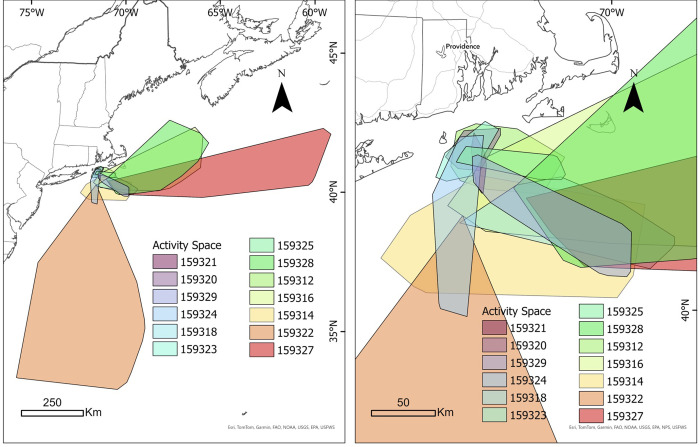
Measures of activity space for each of the 13 blue sharks tracked in the present study. The individual IDs for activity space are ordered and color coded by track length as follows: activity space increases from IDs ordered along the blue to red color range as identified in the figure legend. Similarly, IDs listed earlier in the legend have relatively smaller activity spaces (colder color palate) and those ordered towards the end of the list have relatively larger activity spaces (warmer color palate). Left display shows broader latitudinal range, whereas the right panel is zoomed in on those tracks from the core area off New England. Map developed in ESRI ArcGIS 10.3.3. Reprinted from Esri under a CC BY license with permission from Esri, original copyright 2016.

**Fig 3:**
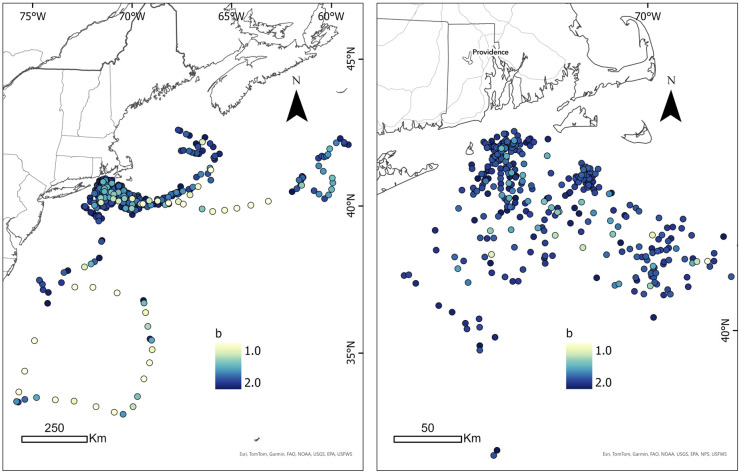
Measures of behavioral state for each interpolated point along each of the tracks for the 13 blue sharks tracked during the present study. Darker color shades represent values closer to 2, suggesting a “resident” state; whereas lighter color shades approaching 1 suggest a “transient” state. Left display shows broader latitudinal range, whereas the right panel is zoomed in on those tracks from the core area off New England. Map developed in ESRI ArcGIS 10.3.3. Reprinted from Esri under a CC BY license with permission from Esri, original copyright 2016.

A total of 13 male blue sharks were tagged with fin-mounted SPOT satellite tags and tracked between of 12–82 days (Table 1, Fig 1). Only male blue sharks were encountered during sampling (sex-specific aggregations are not uncommon in the region), and all sharks were of a similar size class suggesting maturity; the average body length was 264.0 ± 21.0 cm (mean ± SD; cm, total length). TAG ranged from 0.02–0.95 mmol/L, with a mean of 0.30 ± 0.30 mmol l^–1^. Body condition ranged from 0.89–1.09 (0.98 ± 0.06). The total distance traveled for all sharks combined was 9,511 km across a total of 455 tracking days (Fig 1a), with an average rate of movement of 18.49 ± 11.99 km/day across this period. The total activity space of all sharks tracked across the analysis period was 592,624 km^2^ (Fig 2a). The mean behavioral state exhibited by all tracked sharks throughout the sampling period was 1.74 ± 0.23 (Fig 3a), suggesting an overall resident state, whereas calculated tortuosity values averaged 0.70 ± 0.10, suggesting mostly linear movement.

**Fig 4:**
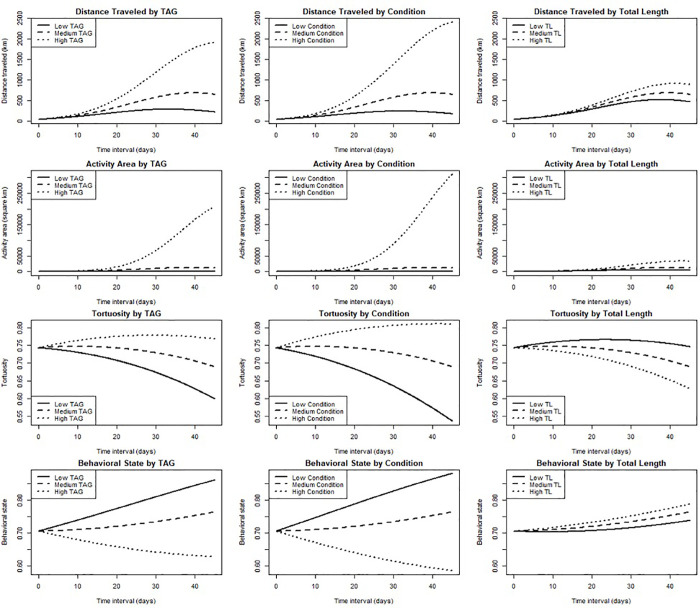
Regression plots showing the modeled relationships between distance traveled, activity area [space], tortuosity, and behavioral state with triglycerides (TAG, mmol^-1^), body condition, and total length (TL, cm), as a function of time at-liberty, for satellite-tagged blue sharks. Lines are drawn following the regression equation as described in the Methods, corresponding to “low, medium, or high” levels of TAG, body condition, or TL (while keeping the other 2 constant). A “low” value is 2 standard deviations below the mean, a “high” value is 2 standard deviations above the mean, and a “medium” value is the mean value for TAG, condition, or TL.

When controlling for elapsed track time, distance traveled was positively and significantly influenced by TAG (*t* = 5.38, *p* < 0.001) and condition (*t *= 6.02, *p* < 0.001) while keeping constant the other variable, with the overall model explaining 79% of the variation (F_5,84_ = 66.17, *p* < 0.001; Table 2, Fig 4). Using the same modeling approach, activity space was positively and significantly influenced by TAG (*t *= 5.37, *p* < 0.001) and condition (*t *= 5.61, *p* < 0.001) while keeping constant the other variable, with the overall model explaining 65% of the variation (F_5,84_ = 30.72, *p* < 0.001; Table 2, Fig 4). Across all 3 of these models, we consistently observed that both TAG and body condition had strong positive interacting effects with time elapsed since capture (Table 2, Fig 4). Total length (TL) also had a positive interaction effect on these responses, but its effect was substantially smaller than either TAG or body condition, and consistently insignificant across all LMs (Table 2).

**Table 2:** Results from models evaluating the relationships between movement metrics and triglycerides (mmol^-1^, TAG), body condition, and total length (cm, TL) in satellite-tracked blue sharks, after controlling for time elapsed. Data for TAG, body condition, and TL were bounded and centered, as were tortuosity and behavioral state (see Materials and Methods). Linear models on log-transformed data were used for distance traveled and activity space, and generalized linear models were used for tortuosity and behavioral state.

Model	Effects	Coefficient	t-value	*p*
*Distance Traveled*	Time elapsed	0.150	8.408	<.001
	Time elapsed^2	−0.002	−5.078	<.001
	Time elapsed:TAG	0.012	5.382	<.001
	Time elapsed:Condition	0.015	6.020	<.001
	Time elapsed:TL	0.004	1.477	0.143
*Activity Space*	Time elapsed	0.241	4.968	<.001
	Time elapsed^2	−0.003	−3.036	<.01
	Time elapsed:TAG	0.033	5.369	<.001
	Time elapsed:Condition	0.037	5.615	<.001
	Time elapsed:TL	0.012	1.855	0.067
*Tortuosity*	Time elapsed	0.005	0.226	0.821
	Time elapsed^2	0.000	−0.548	0.584
	Time elapsed:TAG	0.004	1.771	0.077
	Time elapsed:Condition	0.007	2.629	<.01
	Time elapsed:TL	−0.003	−1.148	0.251
*Behavoiral State*	Time elapsed	0.001	0.042	0.966
	Time elapsed^2	0.000	0.286	0.775
	Time elapsed:TAG	−0.007	−2.671	<.01
	Time elapsed:Condition	−0.009	−3.124	<.01
	Time elapsed:TL	0.002	0.511	0.609

The beta regression model for tortuosity over the elapsed track time showed a significant (positive) effect of only body condition (*z* = 2.63, *p* < 0.001), suggesting that after controlling for all independent variables, every one-unit increase in condition was associated with a 0.007 unit increase in tortuosity (i.e., higher path complexity, Table 2, Fig 4). The beta regression model for behavioral state showed significant effects of TAG (*z* = −2.71, *p* < 0.01) and condition (*z* = −3.12, *p* < 0.01), suggesting that when TAG and condition were each interpedently increased by one-unit, there were decreases in the logit of behavioral state (i.e., a trend towards ‘transient’ behavior) by 0.007 and 0.009, respectively, after controlling for all independent variables (Table 2). Neither tortuosity nor behavioral state exhibited statistically significant relationships with time internal or its square, implying that these are temporally scale-invariant phenomena (Table 2). Animal size did not show a significant effect in any of the models across each of the movement metrics (Table 2).

## Discussion

Understanding the physiological drivers of behavioral plasticity in open ocean fishes offers a pathway to link individual condition with variable patterns of movement and distribution. A recent meta-analysis explored whether physiological traits predicted activity, exploration, and dispersal, revealing effects of metabolism and locomotory performance, which were especially strong in birds and amphibians [30]. Our results show that physiological metrics were associated with variation in short-term (up to 45 days) movement dynamics of blue sharks in the open ocean. This conclusion was supported by the observed correlative effects between stored energy and body condition on both distance traveled and activity space, as well as behavioral state (which were independent of tracking duration).

**Fig 5:**
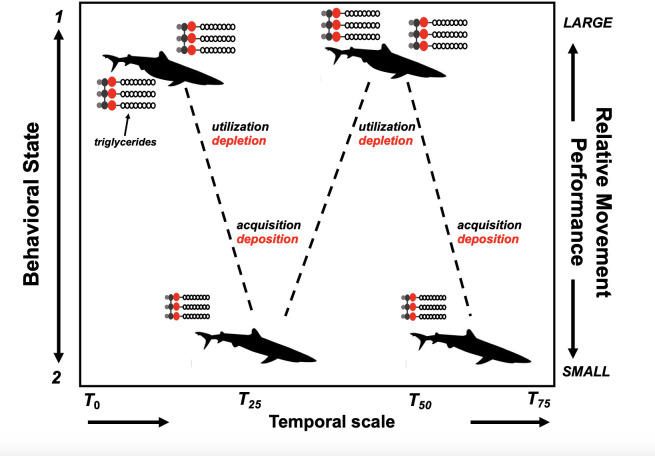
Conceptual diagram hypothesizing metabolic strategy of highly mobile, open-ocean sharks. Triglyceride (as seen in the molecular structures) energy reserves are stored at high levels ( larger molecules suggesting higher levels) and should reflect elevated condition (sharks on top have greater ‘girth’) and should be utilized and depleted over a period of weeks. Sharks with higher energy may remain in transient states at longer periods, whereas those with lower energy may switch to a more ‘resident’ state, whereby individuals may search to acquire prey resources to rebuild energy stores, thus preparing for the next phase of their movement.

The total distance traveled, and activity space covered, by blue sharks in our study showed positive relationships with plasma triglycerides when controlling for total elapsed track time on an individual basis (Fig 4), with these models explaining a relatively high (64–79%) proportion of the variation. Taken together, these results suggest that higher levels of liver-derived lipid stores may increase swimming distance and activity space in open-ocean sharks for periods of up to a month and a half. Given that animals will expand their ranges or move between distant patches when local resources are depleted or if resources are improved in other areas, it is possible that blue sharks which have recently exploited a feeding patch, thus exhibiting higher triglyceride levels, will subsequently be able to undertake more dispersive movements and or exploit distant resource patches (see Fig 5 for a conceptual diagram of these relationships). Indeed, plasma TAG have been correlated with endurance-flight in migratory bird species [47], whereby individuals will ‘fatten-up’ before long periods of flight or re-fuel at known ‘stop-over’ sites [48]. While TAG could, in theory, reflect prior foraging success, this is unlikely in our case as sharks were encountered at the start of the seasonal aggregation, following their northward migration to the shelf where feeding is expected to occur. We therefore interpret elevated TAG as indicative of capacity for future movement, while acknowledging that it may also integrate aspects of recent energetic history.

When fed lipid-rich diets, large open ocean sharks also increase their body condition (e.g., girth relative to length) as these metabolites are deposited in liver tissue [26]. However, body condition is more of a whole-body proxy of overall health accumulated over longer periods than circulating lipid metabolites [49,50,23]. Indeed, body condition has been linked to health and internal energy stores in some species of shark [27]. Body condition was also positively associated with distance traveled and activity space in tagged blue sharks, perhaps reflecting an important dynamic between stored energy and the capacity to move or the ability to take advantage of distant resource patches. Alternatively, but not mutually exclusive, individuals exhibiting higher body condition need not be constrained by local food resources and may search for more profitable resource patches.

Behavioral state analyses of tagged blue sharks suggested that animals with higher energy reserves at the time of capture exhibited more ‘transient’ behavior and more directed movements over the tracking period. We interpret this result to be reflective of a metabolic benefit of stored energy, whereby endogenous energy stores were metabolized to power directed movement to take advantage of (or to search for) more distant areas/resources (Fig 5). Conversely, those individuals with lower energy at the time of capture may not have sufficient energy reserves to exploit distant resource patches and thus show more restricted, convoluted movements, in search of local resources. Yet, behavioral switching may indeed be part of the strategy for these species in the open ocean, as previous data suggest that pelagic sharks periodically adjust their behavioral state during extensive oceanic movements. Indeed, previous research using satellite tags to examine the movements of blue sharks demonstrated behavioral switching from ‘transiency’ to ‘residency’ within the time frame of 20–30 days [40,51]. Moreover, other research demonstrated that blue sharks exhibited frequent ‘knifing’ surfacing behavior, thought to be linked to prey searching [off Ireland, 42]. Work has shown that blue sharks seek out mesoscale oceanographic features (e.g., anticyclonic eddies), possibly to take advantage of deep, mesopelagic prey [off northastern USA, 12,13], with a similar marked switching of modeled behavioral states. Variation in biological oceanography and associated prey fields clearly play a role in determining the decision-making of oceanic species, and these features (e.g., mesopelagic prey, deep scattering layers), if exploited, may likely interact with internal energetic state. It is noteworthy that neither time interval nor its square were statistically significant in the tortuosity and behavioral state models (Table 2). This is unsurprising for behavioral state, which should not necessarily be expected to vary with time, and tortuosity, which was estimated using fractal dimension and is thus by definition scale-invariant [44,46].

Due to the correlative nature of our study and the somewhat limited information on the dyanmics of energy metabolism in large, free-ranging sharks, there are a few important assumptions which deserve transparency. First, plasma triglyceride concentrations depend on a number of factors such as season, maturity status, and gender [29]. However, all tagged sharks were males of similar length and sampled at the same location during the same time of year (summer). It should be noted that although we did not test for the effects of temperature on movement in this study, all sharks were tagged and tracked during the summer season, and the variation in sea-surface temperature was less than 4°C for the temporal period where the majority of individuals (11 out of the 13 blue sharks) were tracked (National Oceanic and Atmospheric Administration 2016). All sharks were also captured using handlines with negligible hook times (< 3 minutes), and were landed instantly, thus reducing potential stress-related effects on energy mobilization. Additional replication and increased satellite track lengths will help to further test the relationships we explored here. We also attempted to mitigate differences of individual tracking duration on our study by standardizing each analysis to a discrete temporal widow. Yet, despite our somewhat limited sample of sharks, we were able to quantitatively ascribe a large proportion of the variation (>75% in many cases) in movement metrics and behavior to a small set of three metabolic variables. Ketone bodies, which were not sampled here, are preferentially oxidized in muscle tissue relative to lipids [24], and as such they may be an important source of metabolic fuel for sharks which spend a significant portion of their lives in the open ocean [17]. Therefore, additional metabolite assays on parameters such as ketone bodies should be considered in future studies to look at differences in metabolic strategies in relation to triglyceride usage across a greater sample of sizes and species. We recognize that snapshot measures of physiology are challenging for pinpointing a specific element of an animal’s life history or any associated physiological mechanisms, and innovations to allow for repeated sampling of blood or metabolic state while an animal is being tracked (using bio-logging devices fitted with probes and sensors) are an important consideration for future work. Furthermore, as the omics toolbox expands, assessing relationships between gene expression and movement behavior should become increasingly common [e.g., 52] and will represent a new frontier in animal performance studies in the wild. As with such studies, including our own here, we caution that the relationships revealed are indeed correlative and we were unable to experimentally test the physiological mechanisms driving variability in movement behaviors.

## Conclusions

Assessments of the individual physiological state have been surprisingly underrepresented in movement studies of marine fishes despite their potential in explaining patterns of variation. A primary result from this study stems from the inferred influence of triglycerides and body condition on the short-term movements of migratory, oceanic sharks obtained by satellite tag telemetry [28,51,53–62]. Our results suggest that variation in these metrics may be important for determining the scale and type of movement of large pelagic consumers, and as such future studies integrating physiology and behavior may help explain variable dynamics resulting from short-term, partial, and long-term movements in tracked animals.

## Supporting information

S1 Data Blue shark Data. (CSV)
